# Genome-Wide Analysis of the Expansin Gene Family in *Populus* and Characterization of Expression Changes in Response to Phytohormone (Abscisic Acid) and Abiotic (Low-Temperature) Stresses

**DOI:** 10.3390/ijms24097759

**Published:** 2023-04-24

**Authors:** Zhihui Yin, Fangwei Zhou, Yingnan Chen, Huaitong Wu, Tongming Yin

**Affiliations:** Key Laboratory for Tree Breeding and Germplasm Improvement, Southern Modern Forestry Collaborative Innovation Center, College of Forestry, Nanjing Forestry University, Nanjing 210037, China; zhihuiyin@njfu.edu.cn (Z.Y.); zhoufangwei@njfu.edu.cn (F.Z.); chenyingnan@njfu.edu.cn (Y.C.); tmyin@njfu.com.cn (T.Y.)

**Keywords:** expansins, *Populus*, phytohormone stress, abiotic stress, gene expression

## Abstract

Expansins are a group of cell wall enzyme proteins that help to loosen cell walls by breaking hydrogen bonds between cellulose microfibrils and hemicellulose. Expansins are essential plant proteins that are involved in several key processes, including seed germination, the growth of pollen tubes and root hairs, fruit ripening and abscission processes. Currently, there is a lack of knowledge concerning the role of expansins in woody plants. In this study, we analyzed expansin genes using *Populus* genome as the study target. Thirty-six members of the expansin gene family were identified in *Populus* that were divided into four subfamilies (EXPA, EXPB, EXLA and EXLB). We analyzed the molecular structure, chromosome localization, evolutionary relationships and tissue specificity of these genes and investigated expression changes in responses to phytohormone and abiotic stresses of the expansin genes of *Populus tremula* L. (PtEXs). Molecular structure analysis revealed that each PtEX protein had several conserved motifs and all of the PtEXs genes had multiple exons. Chromosome structure analysis showed that the expansin gene family is distributed on 14 chromosomes. The PtEXs gene family expansion patterns showed segmental duplication. Transcriptome data of *Populus* revealed that 36 PtEXs genes were differently expressed in different tissues. Cis-element analysis showed that the PtEXs were closely associated with plant development and responses to phytohormone and abiotic stress. Quantitative real-time PCR showed that abscisic acid (ABA) and low-temperature treatment affected the expression of some PtEXs genes, suggesting that these genes are involved in responses to phytohormone and abiotic stress. This study provides a further understanding of the expansin gene family in *Populus* and forms a basis for future functional research studies.

## 1. Introduction

Expansins (EXPs) are non-hydrolytic cell wall relaxation proteins that are involved in developmental processes that alter the cell wall in plants. Expansins directly induce the expansion of the cell wall by disrupting non-covalent bonds between cellulose microfibrils and associated matrix polysaccharides [1]. Expansins were first discovered by Cosgrove in 1989 by studying the acid-induced extension of the hypocotyl cell wall of cucumbers [2]. Subsequent studies demonstrated that genes encoding extended proteins expanded rapidly during speciation and formed large gene families in plants that are widely distributed in various plant genomes in the form of gene families [3].

Canonical plant expansins are small proteins of 250–275 amino acids that are thought to have two conserved domains. The N-terminal domain (D1) is a six-stranded double-psi beta-barrel (DPBB) that is characterized by a His-Phe-Asp motif with conserved polar residues. The D1 domain has up to 30% sequence homology with the glycosyl hydrolase 45 family (GH45); however, expansins’ protein do not possess the hydrolase activity of GH45. The second domain (D2; Pollen allerg) contains conserved aromatic amino acids that are suitable for polysaccharide binding. The residues are aligned on the surface of an a-sandwich fold that resembles motifs from family 63 of carbohydrate-binding module domains (CBM63) [4].

Four subfamilies of expansins proteins have been identified based on phylogenetic sequence analysis, specifically, EXPA EXPB EXLA and EXLB, which are recognized in plants [5]. In addition to regulating cell size, different members of the extended protein family are involved in activities including morphogenesis [6,7,8,9,10,11], root hair growth [12,13,14], pollen fertilization [15], fruit softening [16] and role under adversity stress [17,18,19,20]. A genome-wide analysis of expansins previously identified 36 expansin genes in *Arabidopsis* [21]. Similarly, 56 expansin genes were identified in the rice genome [21] and 75 genes in soybeans [22].

The *Populus* genus is comprised of around 30 different species, including poplars, aspens and cottonwoods. *Populus* are widely distributed across the Northern hemisphere and many species and hybrids have been cultivated worldwide [23,24] as they are fast growing and can readily adapt to a wide range of ecological conditions. *Populus* are a key species for use as roadside trees and in protective forests and so have important ecological, economic and social value [24,25,26].

Plant phytohormones are a group of small, simple organic compounds that play a critical role in plant growth, development, and their response to adversity [27,28,29]. Previous studies have demonstrated that external spraying of salicylic acid enhances rust resistance synthesis in poplars [30]. It was also discovered that gibberellin (GA) treatment reduced the number of adventitious roots in wild-type poplars [31]. Abiotic stresses, which encompass heat, cold, waterlogging, drought, salt, metals, and nutritional deficiencies, are pressures that plants experience that are not caused by the natural environment. Abiotic stresses significantly impact the growth, development and productivity of trees [32,33]. *P. cathayana* plants exhibited increased antioxidant activity and reduced growth and photosystem II efficiency under salt stress [34]. Low temperatures can cause various types of physiological harm, including a reduction in photosynthetic rate, an increase in reactive oxygen species (ROS), decreased nutrient absorption, altered membrane transport and reduced nutrient absorption [35]. *P. cathayana* experienced reductions in growth and physiological functions under drought conditions [36].

Previous research studies have explored the structure and function of expansins in several plant species, such as *Arabidopsis* [37,38,39] and rice [40], yet little is known about the role of expansins in *Populus*. Recently, the availability of the poplar genome [23,41,42] and the genome database [43] have enabled the analysis of the expansin gene family.

The aim of this study was to investigate specific factors regarding the expansin gene family in poplar, its expression pattern in different tissues, and the gene expression response to phytohormone and abiotic stress. This research provides a theoretical basis for further understanding of the molecular processes and roles of expansin genes in the growth, development and stress resistance of poplar.

## 2. Results

### 2.1. Identification of Expansin Genes in Populus and Phylogenetic Relationships

Using the conserved amino acid sequences of the DPBB_1 (Pfam: PF03330) and Pollen_allerg_1 (Pfam: PF01357) domains as queries in HMMsearch, we identified 36 expansin family members in the *Populus tremula* L. All of the family members were divided into four subfamilies consisting of 27 EXPA members, three EXPB members, two EXLA members, and four EXLB members. These members were named *PtEXPA1* to *PtEXLB4* based on their chromosomal locations (Table 1). Further analysis showed that the number of amino acid (AA) residues in the proteins ranged from 185 to 596, with an average of 274 residues. The average molecular weight (MW) was 29.88 kD, which ranged from 20.64 to 67.81 kD. The isoelectric point (pI) for the proteins was between 5.26, and 10.22. 6 of the proteins were acidic and 30 were alkaline.

**Figure 1:**
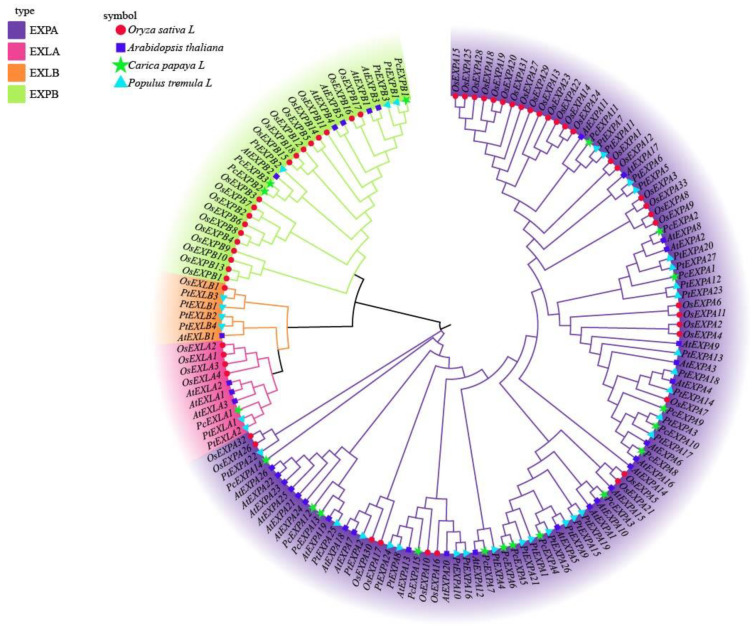
A phylogenetic tree of the expansins from *Populus tremula* L., *Oryza sativa* L., *Arabidopsis thaliana* and *Carica papaya* L. MEGAX was used to construct a Maximum likelihood phylogenetic tree with 1000 bootstrap replications. Circles, squares, stars and triangle represent the expansins of *Oryza sativa* L., *Arabidopsis thaliana*, *Carica papaya* L. and *Populus tremula* L. Purple, pink, orange and green colors represent the EXPA, EXLA, EXLB and EXPB subfamilies.

To study the phylogenetic relationships of expansins, a maximum likelihood phylogenic tree was constructed using MEGAX software based on multiple alignments of 146 expansins from *Populus tremula* L., *Oryza sativa* L., *Arabidopsis thaliana* and *Carica papaya* L. (Figure 1). All of the expansin genes were divided into four subfamilies, specifically, EXPA, EXPB, EXLA and EXLB. The largest subfamily was EXPA and the smallest subfamily was EXLB. 

### 2.2. Gene Structures and Conserved Protein Motifs of PtEXs

**Figure 2:**
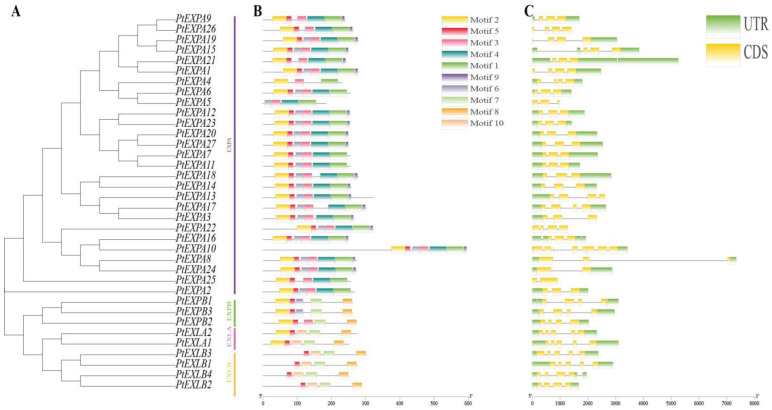
The phylogenetic relationships, exon-intron and motif structures of poplar expansin genes. (**A**) The phylogenetic tree was constructed based on the PtEXs sequences. According to phylogenetic relationships, 36 PtEXs were clustered into PtEXPA, PtEXPB, PtEXLA and PtEXLB groups. (**B**) The 10 motifs of expansin proteins are distinguished by different colors. (**C**) Gene structures of the expansin genes in poplar.

Structural analysis can provide valuable information for the classification of gene evolution events and subfamilies. We analyzed the gene structures and conserved motifs of the expansin family members (Figure 2). Nearly all of the genes had UTR sections at both terminals and the structural patterns of the genes in the same subfamily were similar. Most members of the EXPA subfamily had three or four exons except for *PtEXPA10* (eight exons), *PtEXPA24* and *PtEXPA25* (two exons). The EXPB subfamily had four exons and the EXLA subfamily had five exons. Based on the number of exons, the EXLB subfamily can be separated into two groups with four and five exons, respectively.

Members of the PtEXs gene family had 10 different conserved motifs that are summarized in Supplementary Figure S1 and Supplementary Table S1. The fundamental principle was that the motif composition of the peer group was characterized by the same or similar structure, for example, motif 1,4,9 appeared in nearly all members of the EXPA subfamily. In the other three subfamilies, the motifs were similar and motifs 7 and 8 were conserved in the other three subfamilies. Motif 2 was also found in all three subfamilies of EXPA, EXPB and EXLA, but not in EXLB. These results indicated that the conserved motifs may play critical roles in specific functions, or have similar functionality. Whilst the functions of some motifs were not yet clear, the presence of these conserved motifs reflected functional similarities among the PtEXs.

### 2.3. Chromosome Distributions of the PtEXs 

**Figure 3:**
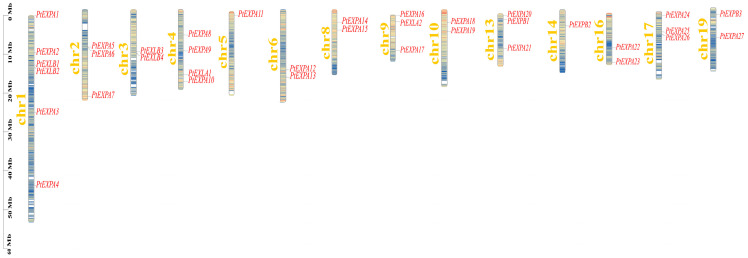
Chromosomal locations of the identified PtEXs in *Populus*. The color gradient from blue to red on the chromosomes indicates the gene density (from low to high).

The chromosomal distribution map of the expansin genes was generated based on the genome data, and 36 expansin genes were unevenly distributed on 14 chromosomes (Figure 3). Chromosomes 7, 11, 12, 15, and 18 did not have expansin genes. Chromosome 1 contained the largest number of expansin genes (up to six), while chromosomes 5 and 14 contain only one expansin gene. The number of expansin genes on the other chromosomes ranged from two to four.

### 2.4. Duplication Events of Expansins

**Figure 4:**
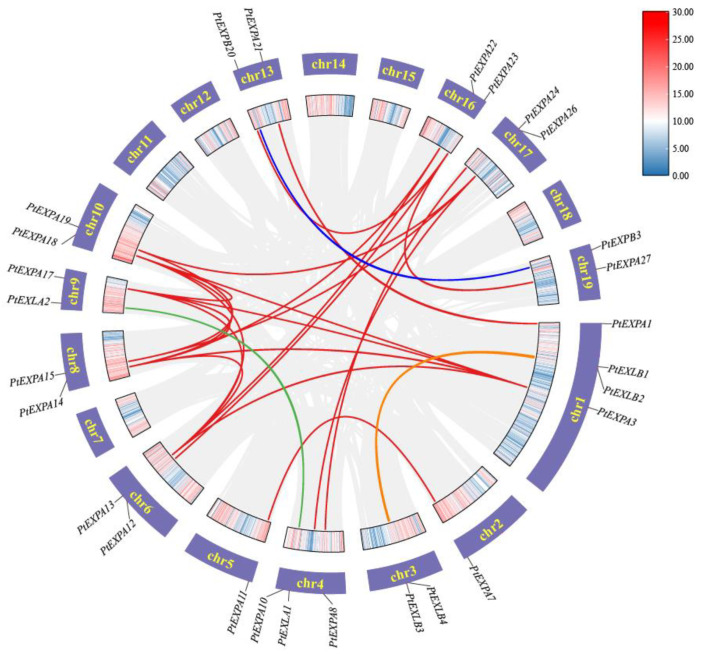
Collinearity mapping of expansin genes in the *Populus* genome. The red, blue, green and orange lines show homologous gene pairs representing the EXPA, EXPB, EXLA and EXLB subfamilies, respectively. From the outside to the inside, the first circle represents chromosome coordinates and the second circle represents gene density distribution.

Many gene families in plants occur as a result of tandem or segment duplications. To better understand the evolution of PtEXs genes, we investigated genome duplication events in this family. The synteny relationship between the PtEXs genes was discovered using MCScanX in TBtools software (v1.108 Chengjie Chen, Guangzhou, China). A total of 20 pairs of 23 genes were obtained and the data were visualized using Circos in TBtools software (v1.108 Chengjie Chen, Guangzhou, China) (Figure 4). The results identified 20 pairs of genes with segmental duplication, suggesting that segmental duplication is the main driver of the evolutionary expansion of the PtEXs gene family. The Ka and Ks of the complex base pairs were calculated using TBtools. The calculated Ks values ranged from 0.220531 to 2.402618, suggesting that replication occurred between 132.01 million years ago and 12.11 million years ago. The Ka/Ks replication gene pairs were less than 1, indicating that the PtEXs gene family was subjected to purification selection during the evolutionary process (Table 2).

### 2.5. Analysis of Cis-Acting Elements

**Figure 5:**
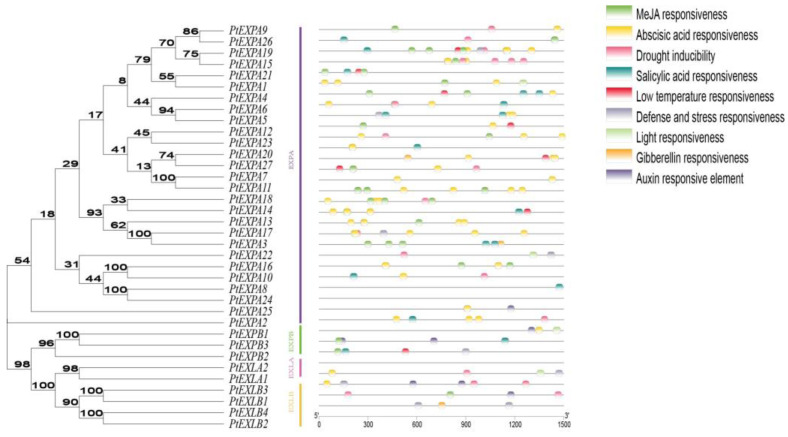
A 1500 bp upstream sequence was used to predict the cis-elements. Each of the 9 predicted cis-elements is represented by a different colored box.

**Figure 6:**
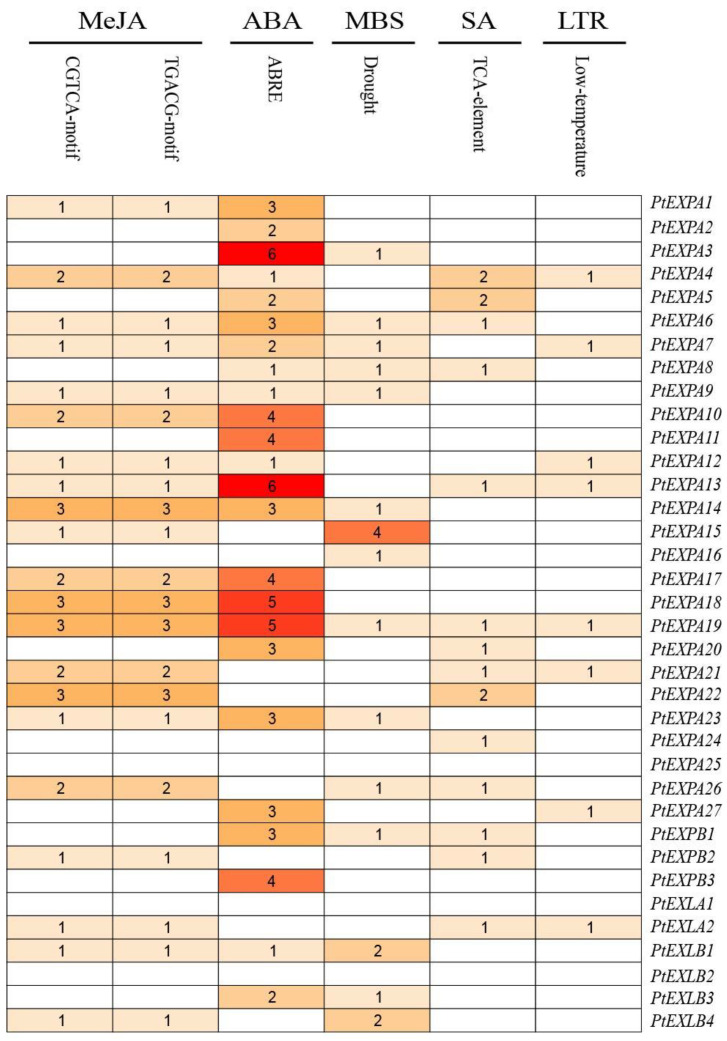
Number of major cis-acting elements of 36 *Populus* expansin genes. The blank box indicates that the quantity was 0. Different colors indicate different number of cis-acting elements.

We extracted the 1500 bp upstream of the 36 PtEXs genes from the initiation codon to study the cis-acting elements of expansin genes (Figure 5). A total of nine cis-acting elements were identified, amongst which Methyl jasmonate (MeJA), salicylic acid (SA), abscisic acid (ABA) and gibberellin (GA) are associated with responses to phytohormone stress, drought-inducible and low-temperature response (LTR), which are abiotic stresses, and light-response, related to plant development. Phytohormone stress and abiotic stress cis-elements were abundant. Further analysis was performed on a number of the five main cis-acting elements (Figure 6). Most of the PtEXs genes contained more ABRE acting elements, and *PtEXPA3* and *PtEXPA13* had the highest number of ABREs, containing six. These data suggest that these genes may play an important role in ABA stress.

Concerning abiotic stresses, we found that some genes with higher ABRE numbers also have cis-acting elements associated with LTR, for example, *PtEXPA13* and *PtEXPA19*. This suggests that the expression of these genes is temperature dependent. We then selected several genes based on the promoter number map for the next step of the analysis.

### 2.6. Analysis of PtEXs Expression in Populus

**Figure 7:**
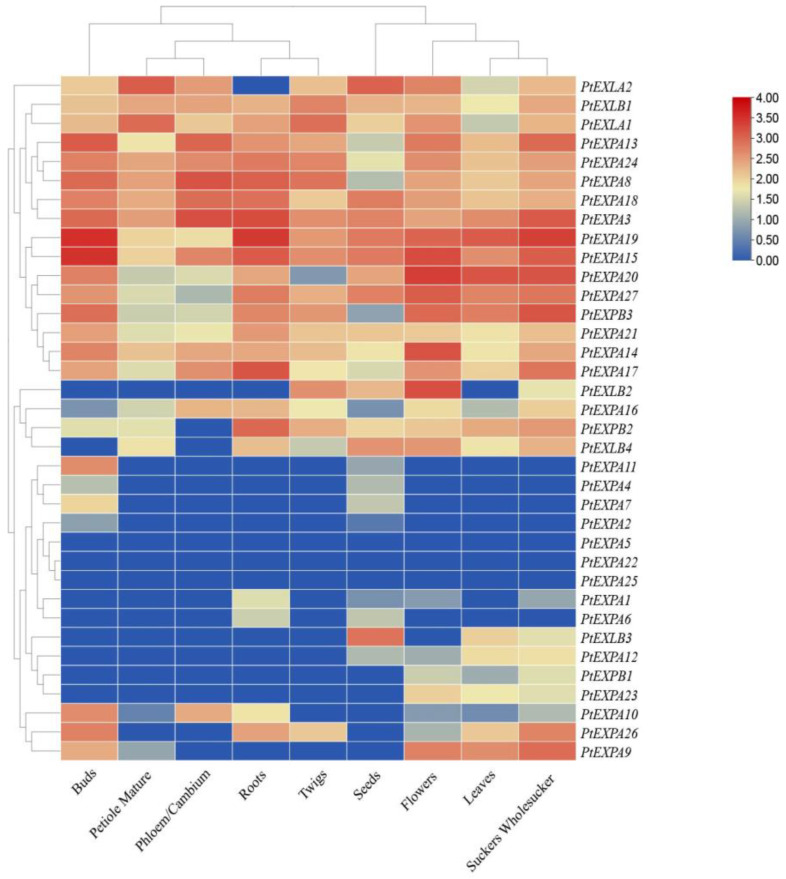
The expression profiles of 36 *Populus* expansin genes in different tissues. The legend represents the logarithmic normalized TPM.

To understand the potential functions of PtEXs gene family members, we downloaded the RNA-seq (TPM values) data through the Popgenie (PlantGenIE.org: Home) website (Supplementary Table S2). The RNA-seq data were used to analyze expression levels in different tissues, including buds, petiole mature, the phloem/cambium, roots, twigs, seeds, flowers, leaves and suckers. The data is presented in the form of a heat map shown in Figure 7. The results showed that different expansin members displayed varying levels of expression in various tissues with differences in transcript levels. *PtEXPA3/8/13/14/15/16/19/20/21/24/27*, *PtEXPB3*, *PtEXLA1* and *PtEXLB1* were expressed in almost all of the tissues. *PtEXPA5/22/25* was not expressed in the tissues. *PtEXPA2/4/7/11* showed similar expression patterns and were only expressed in the buds and seeds, indicating that they may have shared biological processes. *PtEXLB3* was only highly expressed in seeds and largely absent in other tissues, suggesting that it might have a role in seed germination. In general, the expression of the majority of expansin genes varied significantly in buds, flowers, leaves, petiole mature, the phloem/cambium, flowers, roots and seeds.

### 2.7. The Expression of PtEXs in Response to Phytohormone and Abiotic Stresses

**Figure 8:**
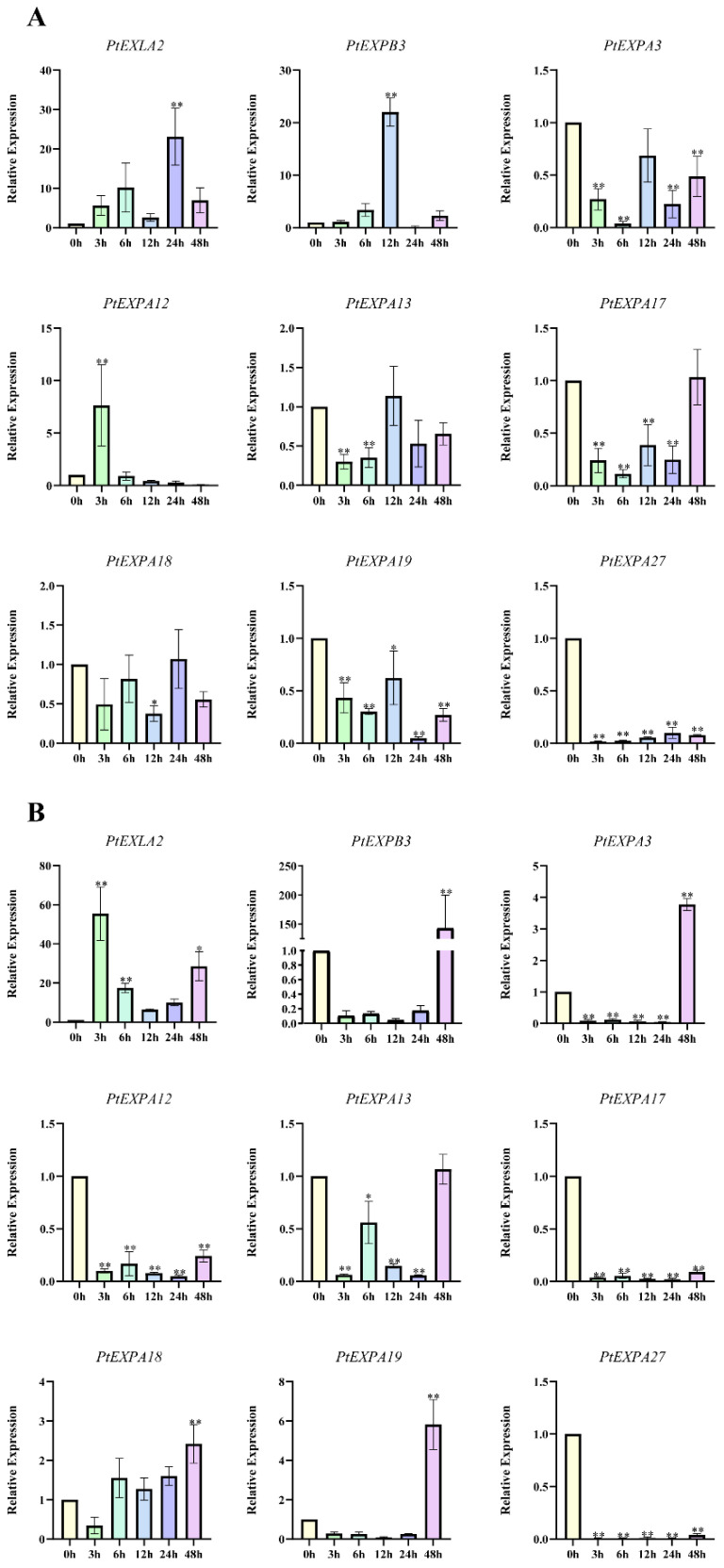
The expression profiles of the 9 PtEXs in 45-day-old *Populus davidiana × P. bolleana* tissue-cultured seedlings. (**A**) ABA and (**B**) low-temperature treatments. The expression of the ubiquitin (UBQ) housekeeping gene was used as a control. The x-axis represents the time and the y-axis represents the level of expression. The data are presented as the mean ± SD (*n* = 3). An asterisk indicates that the expression level after stress was significantly different to the level before the stress (* *p* < 0.05, ** *p* < 0.01).

Given that some phytohormones and abiotic stress-responsive components were identified in the promoter regions of PtEXs (Figure 6), we selected nine expansin genes based on promoter analysis. These genes were abundant in relevant cis-acting elements. For instance, *PtEXPA13/17/18/19* each had at least four ABREs, while *PtEXPA27* and *PtEXLA2* had an LTR. The expression of PtEXs genes at 0, 3, 6, 12, 24 and 48 h after ABA or low-temperature treatments was assessed. Under ABA stress treatment, there were three types of response patterns for PtEXs gene expression. The first expression pattern involved PtEXs genes being induced to express at a high level at a specific time point, followed by a gradual decrease in expression. This pattern includes *PtEXLA2*, *PtEXPB3* and *PtEXPA12*, which were significantly induced to express at high levels at 24 h, 12 h and 3 h, respectively. The second expression pattern category consists of genes significantly repressed by ABA, such as the *PtEXPA27* gene. The third category is the expression pattern without a significant pattern, possibly because these genes were insensitive to ABA treatment. This category includes *PtEXPA3*, *PtEXPA13*, *PtEXPA17*, *PtEXPA18* and *PtEXPA19* (Figure 8A). Similarly, there were three response patterns for PtEXs gene expression following low-temperature treatment. The first category consists of genes induced by low temperature, including *PtEXPB3*, *PtEXPA3* and *PtEXPA19*, all of which were significantly induced to express at high levels after 48 h of treatment. The *PtEXPB3* gene was induced to express more than 150-fold, suggesting that it may be an essential candidate for responding to low temperature. The second category includes genes suppressed by low temperature, such as *PtEXPA12*, *PtEXPA17* and *PtEXPA27*, all of which were significantly repressed after low-temperature treatment. The third category consists of genes with no apparent expression pattern, including *PtEXLA2*, *PtEXPA13* and *PtEXPA18* (Figure 8B).

## 3. Discussion

Plant growth is caused by the proliferation and enlargement of cells and is limited by the cell wall, which restricts the protoplasm. The cell wall restricts the rapid increase of the protoplasm in plant cells, whilst expansin proteins can loosen the cell wall by breaking the hydrogen bonds between cellulose microfibrils and hemicellulose. Expansin proteins play an important role in the growth and development of plants. In this study, we analyzed the *Populus tremula* L. using bioinformatics tools to gain a better understanding of the expasin gene family.

We identified 36 expansin genes with two conserved domains, DPBB_1 and Pollen_allerg_1. The 36 tremula expansins were grouped into four subfamilies, PtEXPA, PtEXPB, PtEXLA and PtEXLB that were similar to other plants. We discovered an uneven distribution of each gene subfamily among species by investigating and comparing the sizes of the expansin subfamilies in *Arabidopsis*, *Oryza sativa*, *Chinese jujube* and other plants (Table 3). Our results showed that EXPA occupied a high proportion of woody plants, suggesting an important role in cell wall regulation. We also found a higher number of EXPBs in non-woody plants compared to woody plants, suggesting that cell wall regulation by EXPB is more effective in non-woody plants.

One of the main mechanisms driving the evolution of genomes and genetic systems is gene duplication [52]. Previous studies have demonstrated that tandem and segmental duplication are the primary forces behind the growth of gene families [53]. A total of 20 pairs of replication genes in the PtEXs gene family were identified that were all segmental. These data suggest that segmental replication is the main driver of the evolutionary expansion of the PtEXs gene family. *Populus* has undergone three genome-wide replication events, namely the ancient replication event (100–120 million years ago), the true rose branch replication event and the *Populus* family replication event (60–65 million years ago) [23]. Recent studies have shown that a whole genome duplication event also occurred in angiosperms 20 million years ago when the temperature and CO_2_ concentration were low. Most of the retained genes were associated with abiotic stresses, such as salt stress, low-temperature stress and drought stress [54]. Our analysis found that *PtEXPA15*/*PtEXPA19*, *PtEXLB1*/*PtEXLB3* and *PtEXPA3*/*PtEXPA17* had 3 pairs of gene duplication times that all coincide with the timing of this Genome-wide duplication event. We hypothesize that these genes play an important role in adversity stress.

Understanding gene expression patterns in tissues is crucial for the mining of functional genes. In previous studies, the expansin genes are expressed in one or more tissues and they are known to be essential for the growth and development of plants. In this study, we analyzed the expression patterns of 36 PtEXs genes in the buds, flowers, leaves, petiole mature, the phloem/cambium, flowers, roots, twigs, seeds and suckers of *Populus tremula* L. Most PtEXs showed tissue-specific expression. *PtEXPA19* and *PtEXPA15* were expressed at higher levels in roots and suckers, suggesting that this gene may play a role in the maturation and development of roots. According to earlier studies, *AtEXP7* and *AtEXP18* can control the initiation of root hairs, in *Arabidopsis*, RNA interference with the expression of *AtEXP7* will result in oppositely shortened root hairs [55], and *GmEXP1* ectopic expression of the gene promoted the growth of transgenic tobacco roots [56]. *PtEXLA1* and *PtEXLA2* were expressed at higher levels in the mature petiole compared to other genes, indicating that the EXLA gene subfamily plays a crucial role in petiole abscission. In *Arabidopsis thaliana*, the altered expression of expansins can modulate the development of leaves and pedicle abscission [7]. *PtEXPA3/8/13/18* were expressed at higher levels in the phloem/cambium, suggesting that these genes may play an important role in wood formation.

Cis-acting elements play a crucial role in transcription and expression in plants [57,58,59]. We evaluated the putative cis-regulatory elements in the 1500-bp putative promoter regions of all of the PtEXs. We found that the promoters of the PtEXs genes mainly contained development-related and adaptation-related elements in response to plant phytohormone and abiotic stress. Previous results have shown that treatment with exogenous auxin after 24 to 48 h increased the expression of one expansin gene (accession no. AF085330) by 50 to 100 fold [60]. Ethylene can also induce the expression of Rp-EXP1 in the leaves of flood-tolerant species [61] and the expression of the LeEXP1 gene is regulated by ethylene in tomato fruits [62]. 

To further investigate the effect of phytohormone and abiotic stress on the expansin gene family, we selected nine genes, based on the number of cis-acting elements, to perform qRT-PCR experiments on ABA and low-temperature stress. We found that the expressions of some PtEXs were increased or repressed by ABA treatment and low temperatures. These experimental results were analyzed in combination with the number of cis-acting elements. We found that *PtEXPA13/18/19* contained five ABREs and were expressed at lower levels following ABA treatment compared to *PtEXPA12*, which has only some ABRE. Similar data were found in response to low temperature stress. *PtEXPB3* with the most significant expression at 48 h did not contain LTR cis-acting elements. Based on these data, we hypothesize that some genes gain new functions during the evolutionary process [63]. Another explanation is that the promoter region may contain cis-elements that enable the gene to respond to low-temperature stress [64].

In conclusion, our data suggest that the Expansin gene family in *Populus* plays an important role in controlling plant physiology and morphology, and has regulatory roles in response to stress. The genome-wide identification and characterization of the expansin gene family members in *Populus* serves as a key foundation for further investigating the function of these genes and may be useful in the breeding and genetic advancement of wood plants. 

## 4. Materials and Methods

### 4.1. Identification of the Expansin Gene Family in Populus

The protein sequence information of poplar was downloaded from the PopGenie database (https://plantgenie.org/ accessed on 5 December 2022) to identify the expansin genes. Utilizing conserved domains of DPBB 1 (PF03330) and Pollen allerg 1 (PF01357) derived from the Pfam database (https://pfam.xfam.org/ accessed on 5 December 2022), a hidden Markov model (HMM) of the expansins was constructed. A search for Hidden Markov Model protein sequences with PF03330 and PF01357 in the *Populus* protein database was performed using HMMsearch with a threshold of e value < 10^−5^ [65]. The results of the initial screening were manually compared with the SMART (Simple Modular Architecture Research Tool) and the NCBI-CDD databases (NCBI conservative domain database) to confirm that the identified members contained both structural domains. For each protein sequence, we analyzed the molecular weight (MW) amino acids (AA) and isoelectric point (pI) on the ExPaSy (https://web.expasy.org/compute_pi/ accessed on 5 December 2022).

### 4.2. Phylogenetic Analysis

To analyze phylogenetic relationships, the expansin protein sequences for *Oryza sativa* L., *Arabidopsis thaliana* and *Carica papaya* L. were obtained from EXPANSIN CENTRAL (http://www.personal.psu.edu/fsl/ExpCentral/ accessed on 12 December 2022). Multiple sequence alignments of the identified *Populus tremula* L., *Oryza sativa* L., *Arabidopsis thaliana* and *Carica papaya* L. expansins were executed using the MEGA X software [66] and a phylogenetic tree was constructed using the Maximum likelihood method. The bootstrap replicates were set to 1000 and all other parameters were left at their default values.

### 4.3. Gene Structure, Chromosomal Locations and Cis-Regulatory Elements

Using the obtained genome annotation data, TBtools was used to map the exon gene structure and chromosome locations [67]. The conserved motifs were identified using the MEME tool (http://meme-suite.org/tools/meme accessed on 15 December 2022). Plant CARE (http://bioinformatics.psb.ugent.be/webtools/plantcare/html/ accessed on 15 December 2022) was used to identify cis-regulatory elements in the 1.5 kb upstream sequences of each expansin gene. The cis-acting elements were visualized using TBtools.

### 4.4. Gene Duplication Analyses

The PtEXs gene family tandem and segmental duplications were investigated using the McscanX in TBtools software (v1.108 Chengjie Chen, Guangzhou, China) [68]. The Advanced Circos function of the TBtools software was used to visualize segmental duplication relationships [67]. TBtools’ simple Ka/Ks calculator was used to calculate Ka/Ks values for collinear pairs. The evolution time (T) was calculated according to the Ks value: T = Ks/2λ, λ = 9.1 × 10^−9^ [63].

### 4.5. Transcriptome Data Analysis

The transcriptional data of PtEXs from 9 tissues/organs in *Populus* were collected from the PopGenie database (PlantGenIE.org: Home) and a correlation heatmap was analyzed using TBtools.

### 4.6. Plant Materials and Treatments

45-day-old *Populus davidiana × P. bolleana* tissue-cultured seedlings were grown by the Key Laboratory of Forest Tree Genetic Breeding, Nanjing Forestry University. The laboratory conditions were as follows: temperature 20 ± 5 °C and substantial daily sunshine (16-h light from 07:00 to 23:00 h, 1000–2000 lx), and 70–80% humidity. Subsequently, we used 36 uniformly growing plants, dividing them into three groups of 12 plants each, with a total of three biological replicates. In each biological replicate, six plants were randomly selected for ABA treatment and low-temperature treatment, respectively. At each of the time points 0, 3, 6, 12, 24 and 48 h after the stress treatment, we collected the second to fourth node leaves of one plant, counting from the top to the bottom. For ABA stress treatment, the plants were fully stressed by mashing the medium. A 100 μM [64,69] ABA solution was poured onto the crushed tissue culture medium so that the plant roots were immersed in the solution. For the low-temperature treatment, the plants were placed in a Percival incubator (Percival, CU-22L) with the temperature set to 10 °C [70,71]. The leaves of the plants were harvested at 6 time points (0, 3, 6, 12, 24 and 48 h) [64,69,72] and immediately frozen at −80 °C.

### 4.7. RNA Extraction and qRT-PCR Analysis

RNA was extracted from the leaves of *Populus davidiana × P. bolleana* using an RNA extraction kit (TIANGEN, Nanjing, China). RNA was detected by 1% agarose gel electrophoresis. RNA was reverse transcribed to synthesize the first strand using a 1-step kit (TIANGEN, Nanjing, China). The cDNA was diluted at a concentration of 1:10. Primers were designed based on the sequences of *Populus davidiana* × *P. bolleana* in the CDS database using Primer 5 software, and the amplification efficiency of each primer pair was analyzed (Supplementary Figure S2 and Supplementary Table S3). The ubiquitin gene (*UBQ*, gene ID Potri.001G418500) was used as a reference control gene [73]. The qRT-PCR reaction was carried out under the following conditions; 1 cycle at 98 °C for 3 min, then 40 cycles at 95 °C for 15 s, 60 °C for 30 s, and 72 °C for 30 s. The 2^−ΔΔCt^ method was used to evaluate genes expression levels [74]. Each sample was analyzed as 3 biological replicates with 3 technical replicates.

### 4.8. Statistical Analysis

For statistical analysis we use GraphPad Prism v8.0.2 software. One-way ANOVA was used to compare the differences between means. Statistically significant difference was considered at * *p* < 0.05 ** *p* < 0.01. The gene expression during 0-h stress treatment was used as a control for significant analysis.

## 5. Conclusions

In this study, we used bioinformatics tools to systematically analyze the expansin gene family in *Populus*. We identified 36 expansin genes that were divided into four subfamilies, EXPA, EXPB, EXLA and EXLB. The gene structure and conserved domain maps were compared according to different families. PtEXs in the same family had similar gene structures and conserved domains. Collinearity, Ka and Ks analysis of the evolution of the PtEXs gene family showed that segmental duplication was the main driving force for the expansion of the genes, and duplicate genes were subject to strong purifying selection in the evolution process. Transcriptome data demonstrated the tissue specific expression of expansins. Combined cis-acting elements and qRT-PCR analysis indicated that some genes may play important roles in stress. Our results will provide a theoretical basis for genetic improvements of the expansin gene family in poplar.

## Figures and Tables

**Table 1:** Summary of information relating to the 36 PtEXs proteins.

	Gene Name	Gene Id	pI	Mw/kD	AAs
EXPA	*PtEXPA1*	Potra2n1c11.1	8.99	30.19	278
EXPA	*PtEXPA2*	Potra2n1c960.1	9.49	29.15	269
EXPA	*PtEXPA3*	Potra2n1c2087.1	9.48	29.02	267
EXPA	*PtEXPA4*	Potra2n1c3505.1	9.69	25.33	231
EXPA	*PtEXPA5*	Potra2n2c4734.1	10.22	20.64	185
EXPA	*PtEXPA6*	Potra2n2c4737.1	8.98	27.76	256
EXPA	*PtEXPA7*	Potra2n2c6293.1	9.1	27.83	256
EXPA	*PtEXPA8*	Potra2n4c9142.1	8.88	30.02	275
EXPA	*PtEXPA9*	Potra2n4c9551.1	8.66	25.81	241
EXPA	*PtEXPA10*	Potra2n4c10239.1	8.77	67.81	596
EXPA	*PtEXPA11*	Potra2n5c10644.1	9.27	27.79	256
EXPA	*PtEXPA12*	Potra2n6c14375.1	9.14	27.62	255
EXPA	*PtEXPA13*	Potra2n6c14588.1	9.62	34.96	323
EXPA	*PtEXPA14*	Potra2n8c17125.1	9.5	2.79	258
EXPA	*PtEXPA15*	Potra2n8c17409.1	9.53	26.72	251
EXPA	*PtEXPA16*	Potra2n9c18642.1	8.09	27.80	250
EXPA	*PtEXPA17*	Potra2n9c19851.1	9.4	33.06	302
EXPA	*PtEXPA18*	Potra2n10c20623.1	9.37	30.40	279
EXPA	*PtEXPA19*	Potra2n10c20953.1	9.9	30.34	279
EXPA	*PtEXPA20*	Potra2n13c24992.1	7.52	26.70	250
EXPA	*PtEXPA21*	Potra2n13c25752.1	9.01	25.59	242
EXPA	*PtEXPA22*	Potra2n16c30169.1	9.34	35.69	323
EXPA	*PtEXPA23*	Potra2n16c30491.1	8.37	27.38	254
EXPA	*PtEXPA24*	Potra2n17c30722.1	8.89	29.94	274
EXPA	*PtEXPA25*	Potra2n17c31117.1	8.69	28.32	257
EXPA	*PtEXPA26*	Potra2n17c31179.1	9.44	28.55	264
EXPA	*PtEXPA27*	Potra2n19c33898.1	6.01	26.54	250
EXPB	*PtEXPB1*	Potra2n13c25121.1	7.61	28.71	262
EXPB	*PtEXPB2*	Potra2n14c26821.1	5.68	28.91	274
EXPB	*PtEXPB3*	Potra2n19c33436.1	8.51	28.60	262
EXLA	*PtEXLA1*	Potra2n4c10039.1	8.25	27.74	252
EXLA	*PtEXLA2*	Potra2n9c18880.1	8.96	29.88	274
EXLB	*PtEXLB1*	Potra2n1c1267.1	5.26	30.20	275
EXLB	*PtEXLB2*	Potra2n1c1318.1	6.57	31.54	290
EXLB	*PtEXLB3*	Potra2n3c7743.1	5.71	33.60	302
EXLB	*PtEXLB4*	Potra2n3c7783.1	6.71	27.61	250

**Table 2:** Ks and Ka analysis of duplicated gene pairs.

Gene 1	Gene 2	Ka	Ks	Ka/Ks	Duplication Type	T (MYA) ^1^
*PtEXLB2*	*PtEXLB4*	0.078312	0.340365	0.230082	Segmental Duplication	18.7
*PtEXPA19*	*PtEXPA15*	0.028716	0.369511	0.077713	Segmental Duplication	20.3
*PtEXLB1*	*PtEXLB3*	0.108542	0.365474	0.296989	Segmental Duplication	20.08
*PtEXPA7*	*PtEXPA11*	0.051896	0.325983	0.1592	Segmental Duplication	17.91
*PtEXLA1*	*PtEXLA2*	0.075097	0.431483	0.174043	Segmental Duplication	23.7
*PtEXPA3*	*PtEXPA14*	0.099992	1.26139	0.079272	Segmental Duplication	69.3
*PtEXPA14*	*PtEXPA17*	0.097609	1.549521	0.062993	Segmental Duplication	85.13
*PtEXPA3*	*PtEXPA17*	0.037618	0.367091	0.102475	Segmental Duplication	20.16
*PtEXPA23*	*PtEXPA27*	0.094301	2.399905	0.039294	Segmental Duplication	131.86
*PtEXPA23*	*PtEXPA12*	0.063563	0.48916	0.129944	Segmental Duplication	26.87
*PtEXPB1*	*PtEXPB3*	0.037749	0.220531	0.171174	Segmental Duplication	12.11
*PtEXPA3*	*PtEXPA18*	0.131003	1.817119	0.072094	Segmental Duplication	99.84
*PtEXPA18*	*PtEXPA14*	0.081269	0.406443	0.199952	Segmental Duplication	22.33
*PtEXPA18*	*PtEXPA17*	0.179824	1.564093	0.11497	Segmental Duplication	85.93
*PtEXPA19*	*PtEXPA26*	0.145877	1.0302	0.141601	Segmental Duplication	56.6
*PtEXPA26*	*PtEXPA9*	0.055452	0.448207	0.123719	Segmental Duplication	24.62
*PtEXPA26*	*PtEXPA15*	0.108133	1.471218	0.073499	Segmental Duplication	80.83
*PtEXPA3*	*PtEXPA13*	0.119706	1.685795	0.071008	Segmental Duplication	92.62
*PtEXPA13*	*PtEXPA17*	0.124216	1.591113	0.078069	Segmental Duplication	87.42
*PtEXPA22*	*PtEXPA14*	0.404278	2.402618	0.168266	Segmental Duplication	132.01

^1^ million years ago (Mya).

**Table 3:** Summary of each expansin subfamily in 10 plant species.

Species	EXPA	EXPB	EXLA	EXLB	Total	Reference
*Populus tremula* L.	27 (75%)	3 (8.3%)	2 (5.5%)	4 (11.1%)	36	In this study
*Arabidopsis*	25 (71.4%)	6 (17.1%)	3 (8.6%)	1 (2.9%)	35	[44]
*Oryza sativa*	34 (58.6%)	19(32.8%)	4 (6.9%)	1 (1.7%)	58	[44]
Chinese jujube	19 (63.3%)	3 (10.0%)	1 (3.3%)	7 (23.3%)	30	[45]
Apple	34 (82.9%)	1 (2.4%)	2 (4.9%)	4 (9.8%)	41	[46]
maize	36 (40.9%)	48 (54.5%)	4 (4.5%)	0 (0%)	88	[47]
Soybean	49 (65.3%)	9 (12.0%)	12(2.7%)	25(20.0%)	72	[48]
Grapevine	20 (69.0%)	4 (13.8%)	1 (3.4%)	4 (13.8%)	29	[49]
Tomato	25 (65.8%)	8 (21.1%)	1 (2.6%)	4 (10.5%)	38	[50]
Tobacco	36 (69.2%)	6 (11.5%)	3 (5.8%)	7 (13.5%)	52	[51]

## Data Availability

Data generated in the current work is provided in the manuscript.

## Supplementary Materials

The following supporting information can be downloaded at: https://www.mdpi.com/article/10.3390/ijms24097759/s1.
