# Supplementary material for: Genome-Wide Analysis of the Expansin Gene Family in Populus and Characterization of Expression Changes in Response to Phytohormone (Abscisic Acid) and Abiotic (Low-Temperature) Stresses
Source: Int J Mol Sci. 2023 Apr 24;24(9):7759. doi: 10.3390/ijms24097759 (PMC10178758; doi:10.3390/ijms24097759)
Supplement: Supplementary file 1 [file ijms-24-07759-s001.zip › ijms-2338070-supplementary.pdf]

Supplementary Material

Supplemental Table S1. Conserved motifs predicted by MEME program in PtEXs.

motif	Width	E-value	Motif sequence
1	50	4.1e-781	RTGWQPMSRNWGQNWQSNNYLNGQSLSFKVTTSDGRTVVSYNVAPSSWSF
2	41	1.8e-664	HATFYGGGDASGTMGGACGYGNLYSQGYGTNTAALSTALFN
3	26	1.8e-438	SDNGGWCNPPRPHFDLAMPMFLKIAE
4	50	7.5e-793	GIVPVSYRRVPCRRRGGIRFTINGHSYFNLVLITNVGGAGDVHAVSVKGS
5	15	1.5e-261	NGLSCGACFEIRCVN
6	21	1.5e-210	SGSPSILITATNFCPPNYALP
7	32	5.60E-98	ELFAYGVVDVEFRRIPCRFPGYNLMFKVHEHS
8	28	9.70E-62	DGKWIWARNVLPEDWKPGMTYDSGVQIT
9	8	7.90E-57	QTFEGKQF
10	29	1.60E-51	DDGVNVVVTDYGEGDNTDFILSTRAYARM

Supplementary Table S2. TPM values of PtEXs genes in transcriptome sequencing of nine poplar tissues.

Gene_id	Buds	Flowers	Leaves	Petiole Mature	Phloem/Cambium	Roots	Seeds	Suckers Wholesucker	Twigs
PtEXPA28	4.96315	4.1698	3.3179	4.074	5.8916	5.3158	5.77575	4.1907	2.9321
PtEXPA25	10.0633	7.18785	8.01635	3.15075	2.1568	9.55545	6.47535	9.55225	4.679
PtEXPA16	5.6102	9.43455	8.00905	1.44115	2.0676	3.8259	4.54045	8.2665	1.2764
PtEXPB2	0	1.57955	1.4232	0.325	0	0.2502	0	2.34805	0.159
PtEXPA23	4.093	3.82725	2.5822	2.4011	2.13285	3.8624	3.20705	3.52345	3.21635
PtEXPB1	2.17315	3.57675	4.84115	2.34695	0	7.0597	3.1399	4.86165	3.6729
PtEXPA2	0	0	0	0	0	0	0	0	0
PtEXPA19	0	2.8448	2.33235	0	0	0	0.23095	2.0261	0
PtEXPA7	5.6326	5.35755	3.7246	4.6936	5.1113	5.9865	3.0788	4.93305	5.09495
PtEXPA3	0	0	0	0	0	0	0	0	0

PtEXPA20	5.6387	1.28375	3.6212	0.3856	0.07525	4.1132	0	5.7772	2.68995
PtEXPB3	6.74765	6.9824	6.26605	1.9664	1.99285	5.44905	1.3299	8.34675	4.5688
PtEXPA17	5.6207	7.59005	6.1199	2.00295	0.78285	5.9735	5.79455	6.853	3.51815
PtEXPA24	0	0.61075	0.11505	0.05515	0	2.25425	0.3636	1.2403	0.499
PtEXLB2	3.37085	3.848	3.02845	4.6962	4.05775	4.19255	3.86825	4.03695	5.6417
PtEXLB3	0.1837	7.9846	0	0.3054	0	0.0831	4.1475	2.27375	4.7496
PtEXPA32	7.0776	4.63425	5.56275	4.8263	7.69545	8.35875	4.6268	7.7714	5.3472
PtEXPA4	0.93515	0.23025	0	0.08745	0	0	0.75065	0.08535	0
PtEXPA1	0.4998	0	0.17095	0	0	0	0.2852	0.05415	0
PtEXPA13	0	0	0	0	0	0	0	0	0
PtEXPA12	0	0	0	0	0	1.0142	1.06055	0.0695	0
PtEXPA14	2.7896	0.0787	0.13175	0	0	0.09165	1.0624	0.03945	0
PtEXLB1	0.27865	0.20205	3.1513	0.20785	0	0.2921	5.85225	2.0942	0.5947
PtEXLB5	0	4.9367	2.613	3.2479	0	3.3525	6.30125	3.83125	1.27005
PtEXLA2	4.04755	4.84995	1.5976	6.84115	3.61765	4.6709	3.3189	3.90135	6.91395
PtEXPA6	4.8876	0.603	0.3407	0.91245	4.21285	2.50185	0.1104	1.4972	0.63915
PtEXPA8	6.9987	5.39455	3.74755	5.33815	7.95215	7.2564	2.92095	5.0085	6.2822
PtEXPA22	4.1977	6.19065	5.7976	1.06965	0.0631	0.0807	0.0757	6.7203	0.76295
PtEXPA15	5.166	0.7039	0	0	0	0.0642	0.70135	0.5274	0.1555
PtEXPA18	0	0.9607	2.62255	0.05925	0	0	1.18215	2.5502	0.05715
PtEXPA27	7.3889	6.40155	4.2291	3.08545	6.3123	5.29515	2.143	7.0422	4.17595
PtEXPA30	5.52195	8.7086	3.3992	3.8161	3.9579	4.10925	3.30105	4.8975	3.8507
PtEXPA26	9.83135	8.6641	5.694	3.2896	5.21195	7.07055	6.4496	7.86615	4.5251
PtEXPA5	2.8184	2.43445	1.9271	2.1785	3.41605	3.45915	1.3122	2.77135	2.53985
PtEXLA1	3.18435	5.59525	1.50705	7.33715	4.99605	0.5511	7.30765	2.95895	3.03725
PtEXPA31	3.8848	4.98595	3.6256	2.38045	4.94465	7.3377	1.76715	6.3342	2.4432

Supplemental Table S3. List of primer sequences used in qRT-PCR reactions

Gene name	Forward Primer	Reverse Primer	Primer amplification efficiency
UBQ	GTTGATTTTTGCTGGGAAGC	GATCTTGGCCTTCACGTTGT	101%
PtEXLA2	GCTGGATGTGGTGCTTGTTT	TGTCTTGACCCATGCCCTTG	101.9%
PtEXPB3	TGAACTCCCAGTCATTTATCG	TCTAGCCACTCAGTGCCTCC	103.3%
PtEXPA3	ACTCAGGCAGCCCGTCTATT	GGAATCCGTTTATTGTGAACCT	102.5%
PtEXPA12	GCCATTCCAGTACCATCACC	ACAGACACCGCACGAACATC	102.7%
PtEXPA13	TGTAACCCACCTCGTCCTCA	CGGAAACCGTTGATAGTGAA	104.3%
PtEXPA17	TTGATGTGGAAGGGTGAAGG	GTCAGCACCACCGTAGAAGG	105%
PtEXPA18	ATCCTCCTCGTCCTCACTTC	TTCACGCTCACTTTCACAATA	105.6%
PtEXPA19	GGGTCTTGCTACGAGATTAGG	AAAGTGTTGTTGAGGAGGGTT	101.5%
PtEXPA27	AGGGAGGGATTAGATTTACCA	GATAGGCTTTGACCGTTGAG	105.3%

.

Supplementary Figure S1. The sequence logos of motifs in PtEXPs protein.

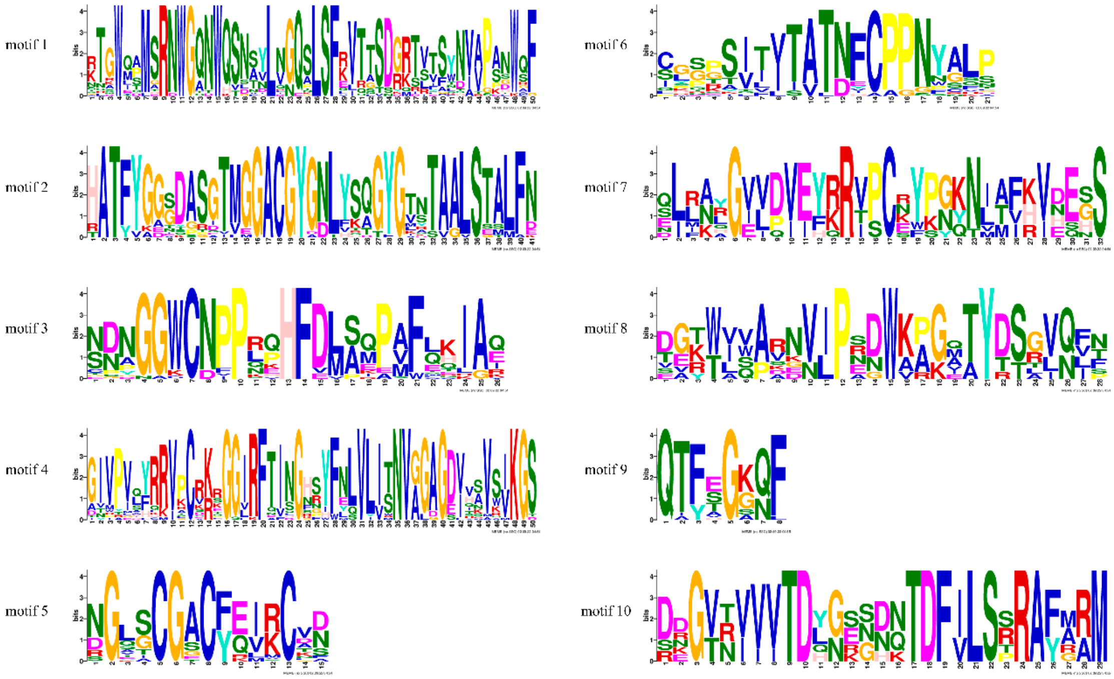

Supplementary Figure S2. Regression line graph on primer amplification efficiency.

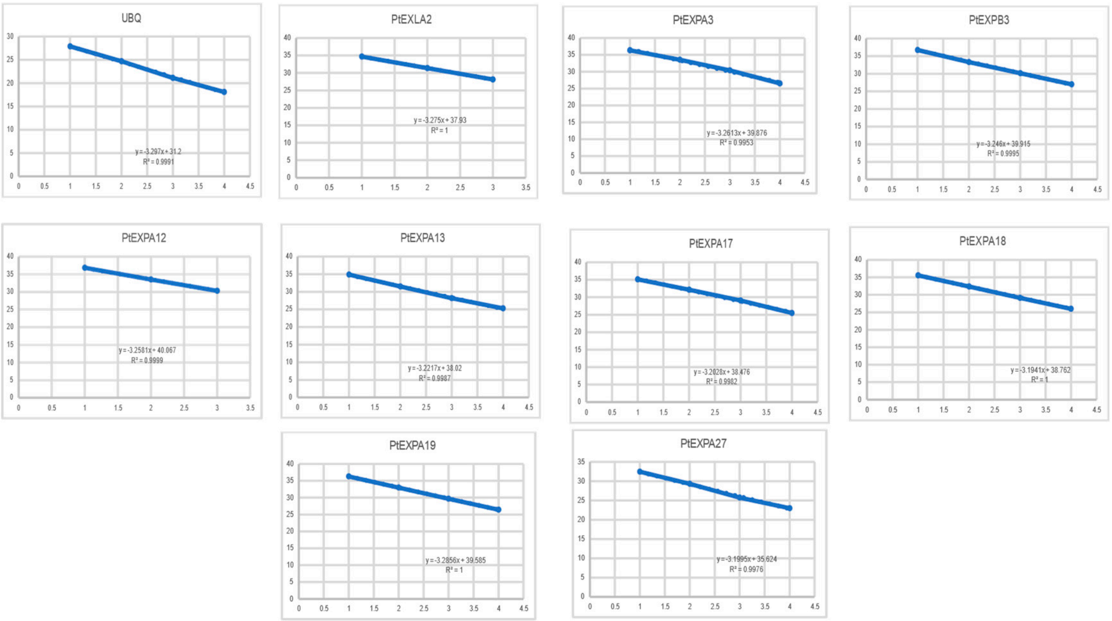

Regarding the amplification efficiency of the primers, the cDNA stock solution treated at low temperature for 0h was subjected to four gradient dilutions of 1×, 10×, 100× and 1000×. The

amplification efficiency of each primer pair was obtained by combining the dominant equation of the regression between the CT value of amplification and the dilution gradient (E=(10^(-1/Slope)-1)%).As PtEXLA2 and PtEXPA12 were not expressed in 1000×, the amplification efficiencies for these two genes were calculated based on three concentration gradients.
